# Association Between Free-Living Sit-to-Stand Transition Characteristics, and Lower-Extremity Performance, Fear of Falling, and Stair Negotiation Difficulties Among Community-Dwelling 75 to 85-Year-Old Adults

**DOI:** 10.1093/gerona/glac071

**Published:** 2022-03-21

**Authors:** Antti Löppönen, Laura Karavirta, Kaisa Koivunen, Erja Portegijs, Taina Rantanen, Taija Finni, Christophe Delecluse, Evelien Van Roie, Timo Rantalainen

**Affiliations:** Faculty of Sport and Health Sciences and Gerontology Research Center, University of Jyväskylä, Jyväskylä, Finland; Department of Movement Sciences, Physical Activity, Sports and Health Research Group, KU Leuven, Leuven, Belgium; Faculty of Sport and Health Sciences and Gerontology Research Center, University of Jyväskylä, Jyväskylä, Finland; Faculty of Sport and Health Sciences and Gerontology Research Center, University of Jyväskylä, Jyväskylä, Finland; University of Groningen, University Medical Center Groningen, Center for Human Movement Sciences, Groningen, The Netherlands; Faculty of Sport and Health Sciences and Gerontology Research Center, University of Jyväskylä, Jyväskylä, Finland; Faculty of Sport and Health Sciences and Neuromuscular Research Center, University of Jyväskylä, Jyväskylä, Finland; Department of Movement Sciences, Physical Activity, Sports and Health Research Group, KU Leuven, Leuven, Belgium; Department of Movement Sciences, Physical Activity, Sports and Health Research Group, KU Leuven, Leuven, Belgium; Faculty of Sport and Health Sciences and Gerontology Research Center, University of Jyväskylä, Jyväskylä, Finland

**Keywords:** Chair rise, Functional performance, Geriatric assessment, Physical function, Physical performance

## Abstract

**Background:**

Good sit-to-stand (STS) performance is an important factor in maintaining functional independence. This study investigated whether free-living STS transition volume and intensity, assessed by a thigh-worn accelerometer, is associated with characteristics related to functional independence.

**Methods:**

Free-living thigh-worn accelerometry was recorded continuously for 3–7 days in a population-based sample of 75-, 80-, and 85-year-old community-dwelling people (479 participants; women *n* = 287, men *n* = 192). The records were used to evaluate the number and intensity (angular velocity of the STS phase) of STS transitions. Associations with short physical performance battery (SPPB), 5-times-sit-to-stand test (5×STS), isometric knee extension force, self-reported fear of falls, and self-reported difficulty in negotiating stairs were also assessed.

**Results:**

The number of STS transitions, mean and maximal angular velocity were lower in older age groups (*p* < .05). All variables were higher in men than in women (*p* < .001) and were positively associated with SPPB total points, knee extension force (*r* ranged from 0.18 to 0.39, all *p* < .001) and negatively associated with 5×STS (*r* = −0.13 – −0.24, all *p* < .05), lower extremity functional limitations (*p* < .01), fear of falls (*p* < .01), and stair negotiation difficulties (*p* < .01).

**Conclusions:**

Free-living STS characteristics were related to lower-extremity performance, lower extremity functional limitations, self-reported fear of falls, and stair negotiation difficulties, which can be a sensitive indicator of impending functional decline. Moreover, STS transitions may provide an indicator of adequacy of lower-limb muscle strength among older individuals.

Sit-to-stand (STS) transitions are one of the most common activities of daily life (1) and good STS performance is an important factor in maintaining functional independence (2). STS transitions challenge the older adult’s balance and might be a cause of falls when the ability to transfer from STS is limited (3,4). Usually, STS transitions assessment is based on laboratory measurements, for example, using 5-times-sit-to-stand test (5×STS) (5). However, laboratory-measured capacity should not be equated to functioning in free-living environment when measuring older adults without mobility limitations (6,7), as for example, the full maximal capacity is not always utilized in everyday performance. The weakness of the laboratory measurements is that they do not necessarily indicate performance in free-living environment where human intrinsic capacity and environmental factors affect participation and activities (8,9). Therefore, knowledge of behavior in the free-living environment may be of interest.

The recent miniaturization of technology has made prolonged recordings of free-living physical behavior feasible (10). Inexpensive and wearable tri-axial accelerometers have been shown to reliably distinguish body postures and physical activity types (11–13). Accordingly, accelerometers (typically thigh-worn) have been used to assess STS transitions in the free-living environment (1). Previous studies have quantified the volume of STS transitions (ie, number per day) in the free-living environment using 2 accelerometers (sternum and thigh) (14,15). Moreover, inertial measurement units have been used to estimate STS transition duration and power in a laboratory setting (16,17). Kinematics (angular velocity and vertical velocity) are more indicative of the mechanical requirements of the STS transition than just the time taken to complete the transition and hence could reveal further insight compared to the completion time by enabling evaluation of the manner of completing the transition (18). However, the previous free-living STS transition explorations have typically used transition duration to indicate the intensity of the STS transition rather than evaluating the kinematics directly (14,15).

Pickford et al. (2019) have evaluated STS transition kinematics in the free-living environment using a proprietary algorithm to compare peak velocities of STS transitions between stroke survivors and unaffected peers (19). To the best of our knowledge, there is no publicly available algorithm that can detect STS transitions and quantify the STS transition intensity by kinematics based on free-living thigh-worn tri-axial accelerometer records. Therefore, we developed a new algorithm in the current study to detect and quantify STS transitions (Supplementary Code).

The purpose of the present study was to explore whether detected volume and quantified intensity of free-living STS transitions are associated with lower-extremity performance, self-reported fear of falling, and stair negotiation difficulties among community-dwelling 75-, 80-, and 85-year-old people. Based on previous research, which has indicated that limited STS transitioning performance is associated with difficulties in stair negotiation, weak knee-extensor muscles, and high body mass (20–24), we hypothesized a moderate association between the above mentioned laboratory-based performance characteristics and number and intensity of free-living STS transitions. In addition, 5×STS test has been shown to be associated with the risk of falling (25) which we considered a sufficient justification to hypothesize an association between fear of falling and free-living STS performance.

## Method

### Study Design and Setting

We used data from the AGNES-study (Active Aging―Resilience and external support as modifiers of the disablement outcome; *n* = 1 021), which was conducted in the Gerontology Research Center, the University of Jyväskylä. AGNES comprises three age cohorts (75, 80, and 85 years-of-age) of people living independently in the city of Jyväskylä, in Central Finland. The study protocol has been published by Rantanen et al. (26) and Portegijs et al. (27), and the study was approved by the ethical committee of the Central Finland Health Care District.

AGNES-study participants were asked to participate in laboratory measurements, which (*n* = 782) were used to develop algorithms to detect and quantify free-living STS transitions (Supplementary File). All AGNES-study participants who participated in the laboratory testing were also asked for interest in providing a 3–7 days free-living accelerometry record (*n* = 479), which was used to identify the volume and quantify the intensity of free-living STS transitions. The flow chart of the study has been reported elsewhere (27). The records were obtained between October 2017 and December 2018. Accelerometry was conducted with a thigh-worn accelerometer (tri-axial accelerometer, which sampled continuously at 100 Hz, 13-bit analog-to-digital conversion, acceleration range ±16g, UKK RM42, UKK Terveyspalvelut Oy, Tampere, Finland) taped on using a transparent adhesive film for waterproofing on the anterior aspect of the dominant thigh for 7–10 consecutive days following a home interview. The accelerometers were taped on by a research assistant at participants’ home and removed at the research center.

### Data Processing

The algorithms were developed using Matlab (R2019b, The MathWorks Inc., Natick, MA, USA). In the first phase, the magnitude (Euclidian norm) of the resultant acceleration for each sampling instant was calculated from raw accelerometer data. Mean amplitude deviation (MAD) was calculated in nonoverlapping 5 s epochs based on the magnitude of the resultant acceleration (28).

To identify the instantaneous orientation of the thigh, we calculated an angle for postural estimation (APE) from resultant acceleration values using the method described by Vähä-Ypyä et al. (13). The calculation requires knowing the direction of gravitational pull when the participant is upright (reference vector). This was defined as the median of the mean X, Y, and Z accelerations of each continuous bout ≥20 s with MAD between 0.035 g and 1.2 g. These MAD cutoffs were identified from the AGNES-study laboratory session 6-minute walking test data to include all participants, and hence, bouts with such characteristics comprise walking. During walking, the mean orientation of the thigh is upright, and the median acceleration is equivalent to that caused by the pull of gravity. The instantaneous acceleration in each of the recorded directions was low-pass filtered with a 1 Hz zero-lag Butterworth filter, and APE was subsequently calculated for each time instant as the vector angle between the instantaneous filtered acceleration vector and the reference vector. After that, the APE-signal was smoothed with a 4th-order Butterworth zero-lag low-pass filter with a 10 Hz cutoff frequency. The filtered APE-signal was transferred into a rectangular signal with a value of 1 when APE < pi/4, and a value of 0 otherwise. That is, upright thigh posture was assigned 1, and horizontal 0. This rectangular signal was then smoothed with a sliding median filter of 23 samples to produce the final posture estimation signal. The 23-sample length for the median filter, as well as the 2 (1 Hz & 10 Hz) Butterworth filter cutoff frequencies were selected based on experimentation.

STS transitions were thereafter identified as follows: all posture estimation signal transitions from 0 (horizontal) to 1 (upright) were considered as candidate STS transitions. A candidate was accepted as a STS transition if the following 3 criteria were met: (a) the variance of the magnitude of the resultant acceleration between 2.5 s and 0.5 s prior to the candidate transition was less than 0.02 g (ie, the participant had been stationary for at least 2 s prior to the transition), (b) starting angle of the STS transition (APE signal) was more than 65 degrees (1.14 rad) and, (c) the movement of the transition ended at an angle of less than 35 degrees (0.61 rad). Due to the variance criterion the algorithm will only detect the first of a set of STS movements (eg, if a person did continuous seat-based squatting starting from a seated posture, only the first STS would be included).

The intensity of an identified STS transition was estimated based on the APE-signal time derivative (ie, angular velocity) as follows: baseline APE (corresponds to thigh angle prior to standing up) was established as the mean between 2.5 s and 1.5 s to the identified transition instant. The last sample at the baseline value prior to the transition instant was thereafter set as the initiation of the angular velocity determination. Linear fits were then applied to each data set from the initiation sample until the transition instant to transition instant + 0.15 s with one sample length increment. The longest fit where the square of the last instant of the fit and the APE differed by less than experimentally determined 0.005 degrees was chosen, and the slope of the chosen fit is reported as the STS transition intensity. The STS transitions detection accuracy of the algorithm was examined using the laboratory session of this study. Prior to the 6-minute walk test, the protocol included two known STS transitions that were defined as the ground truth. Ground truth STS transitions identified by the algorithm were defined as true positives. Ground truth STS transitions that could not be identified were defined as false negatives. No false positives were identified, and we did not attempt to define a true negative. True positives and false negatives were used to calculate detection accuracy, which ranged between 82.7% and 97.5% depending on the age (better accuracy among younger age groups compared to older age groups) and sex (better accuracy among men than women) groups, with an overall accuracy of 93.3% (Supplementary File). The angular velocity quantification accuracy of STS transitions at different velocities was good when the angular velocity detected by the algorithm was compared against 2D motion analysis (Supplementary Video). In addition, the volume and intensity of STS transitions monitored by thigh-worn accelerometer are reproducible from day-to-day to year-to-year (29).

The volume of the STS transitions was determined as the number of transitions per monitoring day, and the STS transitions mean intensity (mean median angular velocity) was determined as the mean of daily median transitions. The maximal intensity (maximal angular velocity) was defined as the median of the ten fastest STS transitions over the entire monitoring period. No participant exceeded 4 rad/s in the laboratory, and therefore we filtered out any STS transitions above 4 rad/s from the data prior to the estimation of the maximum free-living angular velocity. A total of 79 transitions were removed due to this, and this was 0.04% of all 182 103 transitions detected in this data set.

### Descriptive Characteristics and Other Measurements

Age and sex were extracted from the population register, body height (stadiometer), body mass (digital scale Seca, Hamburg, Germany), socioeconomical status (self-reported years of education), and cognitive function test (mini-mental state examination, MMSE) were assessed using standardized procedures (26). Lower-extremity performance was assessed in the laboratory (knee extension force) or the participant’s home by the short physical performance battery (SPPB). Maximal knee extension force of the dominant lower limb with the knee at 60 degrees was measured in a sitting position using an adjustable dynamometer chair (Metitur LTD, Jyväskylä, Finland). At least 3 attempts were required, and the highest force was chosen for the analyses (30). The SPPB comprised tests on standing balance, walking speed over a 3-m distance, and the 5×STS (5,31). In this study, we used the SPPB total score (maximum of 12 points, higher scores mean better performance) and the time of the 5×STS test as outcomes. Good lower extremity function was defined as 11-12 SPPB total points and limited lower extremity function as 10-3 SPPB total points (32).

Fear of falling was assessed by the question “Are you afraid of falling?” with 4 response options: never, occasionally, often, and constantly (33). In this study, “never” was categorized as “No Fear,” and the rest of the response options were merged into “Yes Fear.” Difficulties in negotiating stairs were assessed with the question: “Have you noticed any of the following changes in your ability to ascend a flight of stairs?” The responses were categorized as “No difficulties,” “I can ascend a flight of stairs, but I have some difficulties,” “I can ascend a flight of stairs, but I have a lot of difficulties,” “I cannot ascend a flight of stairs without help of another person,” or “I cannot ascend a flight of stairs even with help.” In the present study, “no difficulties” were categorized as “No difficulties,” and the rest of the response options were merged into “Yes difficulties.” No participant reported “I cannot ascend a flight of stairs even with help.” Self-reported habitual physical activity was assessed using the Yale Physical Activity Survey for older adults (8-item). The total score range was 0–137 and higher scores indicate higher physical activity (34).

### Statistical Analyses

Results of STS transitions are reported as mean and standard deviation (*SD*). Associations between variables were tested with Spearman rank correlation coefficients. Number, mean, and maximal angular velocity of STS transitions were categorized into tertiles group comparisons for 5×STS time and knee extension force normalized for body mass. Tertiles (as opposed to quartiles, quintiles, etc.) were selected to maintain sufficient sample sizes in each group. Shapiro-Wilk normality test indicated that some of the variables were not normally distributed, and nonparametric statistical tests were therefore chosen. Sex and age group differences were analyzed with Mann–Whitney U (Wilcoxon rank-sum) test for categorical/dichotomous variables and Kruskal–Wallis test for continuous variables. Tertiles comparisons were performed using the Kruskal–Wallis multiple comparison test and the Dunn’s test (Holm-Bonferroni method) in pairwise comparisons. Sensitivity analyzes between sexes and age groups were performed between the variables and the self-reported questions (fear of falling, lower extremity functional limitations, and difficulties in negotiating stairs). Statistical significance was set at *p* < .05 (2-tailed), and analyses were performed in the “*R*” statistical environment (version 4.1.1) (35).

## Results

Descriptive characteristics and free-living STS transitions of the participants are presented in Table 1. The number of STS transitions, mean and maximal angular velocity differed between age groups and sexes (all *p* < .001). The 85-year-old women showed 19.6% fewer STS transitions (*p* = .005) and 9.2% lower mean (*p* < .001) and 14.6% lower maximal angular velocity (*p* < .001) in the free-living environment compared to the 75-year-old women. The 85-year-old men showed 18.3% fewer STS transitions (*p* = .015) and 8.9% lower mean (*p* = .012), and 9.4% lower maximal (*p* = .042) angular velocity of the STS transitions compared to the 75-year-old men.

**Table 1.** Descriptive Characteristics of the Participants and Results of SPPB, Knee-Extension Force, Volume and Intensity of STS Transitions in Each Age Group (Mean ± Standard Deviation [*SD*])

	75 Years (*n* = 244)		80 Years (*n* = 153)		85 Years (*n* = 82)		*p* Value*		*p* Value^†^
	Women	Men	Women	Men	Women	Men	Ages		Sexes
Variable	*n* = 149	*n* = 95	*n* = 87	*n* = 66	*n* = 51	*n* = 31	Women	Men	
MMSE (points)	27.8 ± 2.2	27.4 ± 2.3	27.7 ± 1.9	27.1 ± 3.0	26.7 ± 2.8	26.5 ± 2.4	.021	.183	.053
Years of education	12.1 ± 4.1	12.3 ± 4.5	11.4 ± 4.1	11.8 ± 3.9	10.3 ± 4.3	10.0 ± 4.9	.003	.022	.454
YPAS (points)	57.8 ± 22.6	61.8 ± 22.6	59.2 ± 20.2	65.3 ± 27.5	50.2 ± 20.6	55.8 ± 24.6	.019	.126	.020
SPPB overall points (points)	10.5 ± 1.6	10.8 ± 1.6	10.4 ± 1.8	10.5 ± 2.0	9.2 ± 2.2	9.7 ± 1.9	.001	.003	.067
Five times STS test time (s)	12.4 ± 3.4	12.2 ± 3.3	12.3 ± 3.5	11.9 ± 4.6	14.1 ± 4.1	13.9 ± 4.8	.029	.048	.301
Knee extension force (N/kg)	4.4 ± 1.2	5.8 ± 1.5	4.2 ± 1.3	5.4 ± 1.4	3.6 ± 1.0	4.8 ± 1.0	<.001	.002	<.001
Number of STS (no/d)	42.8 ± 16.3	50.4 ± 16.8	41.4 ± 15.4	47.3 ± 18.8	34.4 ± 15.2	41.2 ± 14.1	.005	.015	<.001
Mean angular velocity (deg/s)	57.6 ± 8.5	60.6 ± 8.8	56.1 ± 8.4	59.8 ± 9.5	52.3 ± 7.5	55.2 ± 9.0	<.001	.012	<.001
Max angular velocity (deg/s)	109.0 ± 18.8	115.9 ± 20.0	106.5 ± 22.9	112.3 ± 18.6	93.1 ± 14.8	105.0 ± 20.9	<.001	.042	<.001

*Notes:* MMSE = Mini-Mental State Examination; SPPB = Short Physical Performance Battery; STS = sit-to-stand transitions; YPAS = Yale Physical Activity Survey for older adults.

*Independent-samples Kruskal–Wallis test.

^†^Independent-samples Mann–Whitney U test.

The Spearman rank correlation coefficients between variables are given in Table 2. In the number of STS transitions, mean and maximal angular velocity were positively associated with the SPPB total points and maximal isometric knee extension force (*r* ranged from 0.18 to 0.39 all *p* < .001) and negatively associated with the 5×STS test (*r* = −0.13 – −0.24, *p* < .05). We also ran the correlation analyses for sexes and age-groups independently as a sensitivity analysis, and the correlations did not differ markedly between sexes and age groups.

**Table 2.** Spearman’s Correlation Coefficients Between Free-Living Sit-to-Stand Variables, Physical Activity Behavior and Performance Tests

	Number of STS (no/d)		Mean Angular Velocity (deg/s)		Max Angular Velocity (deg/s)	
Variable	*r*	*p* Value	*r*	*p* Value	*r*	*p* Value
Mean angular velocity (deg/s)	0.53	<.001				
Max angular velocity (deg/s)	0.50	<.001	0.65	<.001		
Five times STS test time (s)	−0.13	<.004	−0.18	<.001	−0.24	<.001
SPPB overall points (points)	0.18	<.001	0.24	<.001	0.33	<.001
Knee extension force (N/kg)	0.25	<.001	0.28	<.001	0.39	<.001

*Note:* SPPB = Short Physical Performance Battery; STS = sit-to-stand transitions. *p* Value (2-tailed).

Tertiles group comparisons are presented in Table 3. Overall, the tertiles based on maximal and mean angular velocity of STS transitions demonstrate more differences between tertile groups in laboratory-based 5×STS and knee extension force than tertiles based on number of STS transitions. This indicates that lower STS transition velocities are linked to longer 5×STS time and lower knee extension force. In particular, the weakest tertile (T1) seems to differ from the others, while T2 and T3 do not seem to differ from each other.

**Table 3.** Participants Were Divided into Three Groups, i.e., Lowest, Middle and Highest Tertile, Based on Their STS Performance in the Free-Living Environment (Column 1: Number, Mean, or Maximal Angular Velocity of STS Transitions)

	Women	Men	Women	Men	Women	Men
Tertiles based on number of STS*						
	Number of STS (no/d)		5×STS test time (s)		Knee extension force (N/kg)	
Lowest (T1)	24.5 (6.4)	30.8 (8.0)	12.9 (3.8)	12.6 (3.5)	4.0 (1.2)	5.2 (1.6)
Middle (T2)	39.2 (3.9)	45.9 (3.7)	12.8 (3.8)	13.3 (5.1)	4.3 (1.3)	5.5 (1.5)
Highest (T3)	59.1 (11.0)	67.1 (12.5)	12.3 (3.2)	11.2 (3.2)	4.3 (1.2)	5.8 (1.2)
*p* Value #	<.001^†^^,^^‡^^,^^§^	<.001^†^^,^^‡^^,^^§^	.631	.013^‡^	.107	.013^§^
Tertiles based on mean angular velocity^,^^‖^						
	Mean angular velocity (deg/s)		5×STS test time (s)		Knee extension force (N/kg)	
Lowest (T1)	47.2 (3.1)	49.3 (3.8)	13.1 (3.7)	13.1 (4.1)	3.8 (1.1)	5.0 (1.2)
Middle (T2)	55.9 (2.1)	59.5 (1.9)	13.0 (3.8)	11.8 (3.7)	4.3 (1.3)	5.9 (1.8)
Highest (T3)	65.7 (5.6)	69.6 (5.5)	11.9 (3.2)	12.2 (4.5)	4.5 (1.3)	5.6 (1.2)
*p* Value #	<.001^†^^,^^‡^^,^^§^	<.001^†^^,^^‡^^,^^§^	.026^§^	.078	<.001^†^^,^^§^	.004^†^
Tertiles based on max angular velocity^¶^						
	Max angular velocity (deg/s)		5×STS test time (s)		Knee extension force (N/kg)	
Lowest (T1)	85.1 (8.7)	91.7 (9.1)	13.7 (3.8)	13.6 (4.3)	3.7 (1.1)	4.9(1.3)
Middle (T2)	104.2 (3.8)	112.8 (5.0)	12.5 (3.8)	11.5 (3.2)	4.3 (1.4)	5.5 (1.3)
Highest (T3)	127.3 (16.3)	134.2 (13.4)	11.8 (2.8)	11.9 (4.4)	4.7 (1.1)	6.1 (1.6)
*p* Value #	<.001^†^^,^^‡^^,^^§^	<.001^†^^,^^‡^^,^^§^	<.001^†^^,^^§^	.010^†^^,^^§^	<.001^†^^,^^‡^^,^^§^	<.001^†^^,^^§^

*Notes:* Data (mean [*SD*]) represent values of the different tertiles on lab-based measurements, i.e., 5-times-sit-to-stand test (5×STS time) and knee extension force normalized for body mass. SPPB = Short Physical Performance Battery; *SD* = standard deviation; STS = sit-to-stand transitions.

*Women tertiles cutoff: T1 ≤ 33.17, T3 ≥ 46.00; Men tertiles cutoff: T1 ≤ 40.15, T3 ≥ 52.40.

^†^T1–T2 *p* < .05.

^‡^T2–T3 *p* < .05.

^§^T1–T3 *p* < .05.

^‖^Women tertiles cutoff: T1 ≤ 51.66, T3 ≥ 59.74; Men tertiles cutoff: T1 ≤ 55.32, T3 ≥ 62.67.

^¶^Women tertiles cutoff: T1 ≤ 97.22, T3 ≥ 111.21; Men tertiles cut-off: T1 ≤ 103.15, T3 ≥ 120.80.

^#^Independent-samples Kruskal–Wallis test (Pairwise: Dunn’s test, Bonferroni-Holm).

**Figure 1.**
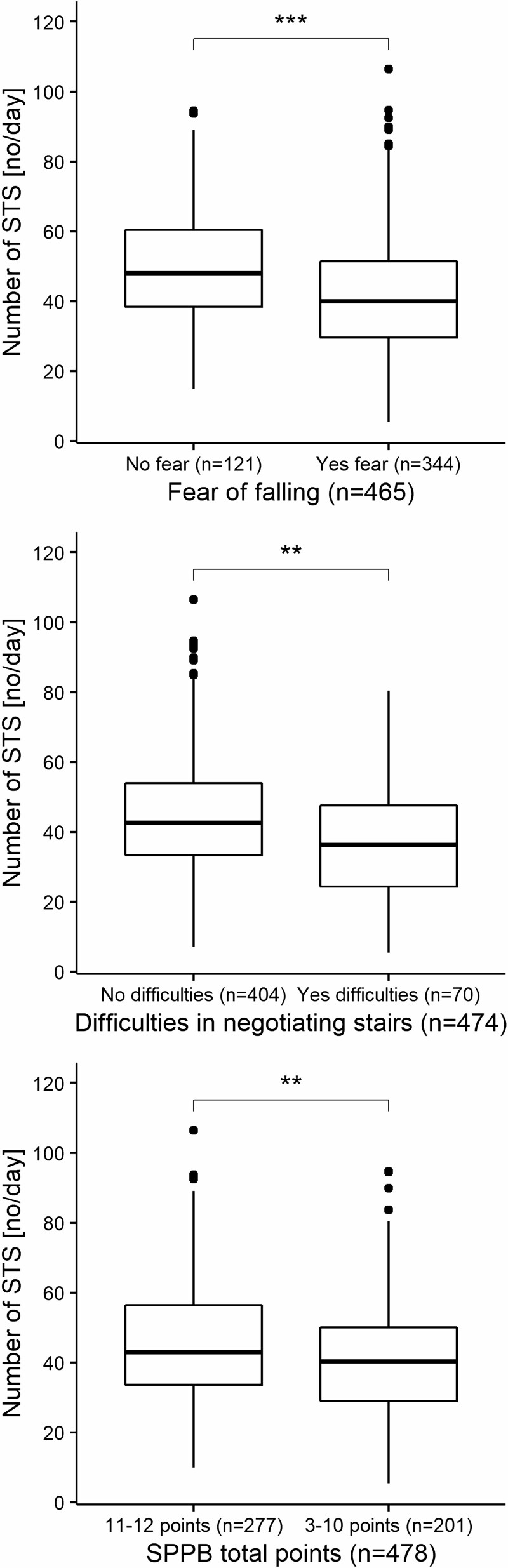
Number of STS transitions group comparisons between self-reported fear of falling, difficulties in negotiating stairs, and lower extremity functional limitations in free-living environment. Independent-Samples (unpaired) Mann–Whitney U Test (Wilcoxon rank-sum). ***p < .001, **p < .01 (2-sided). STS = sit-to-stand.

**Figure 2.**
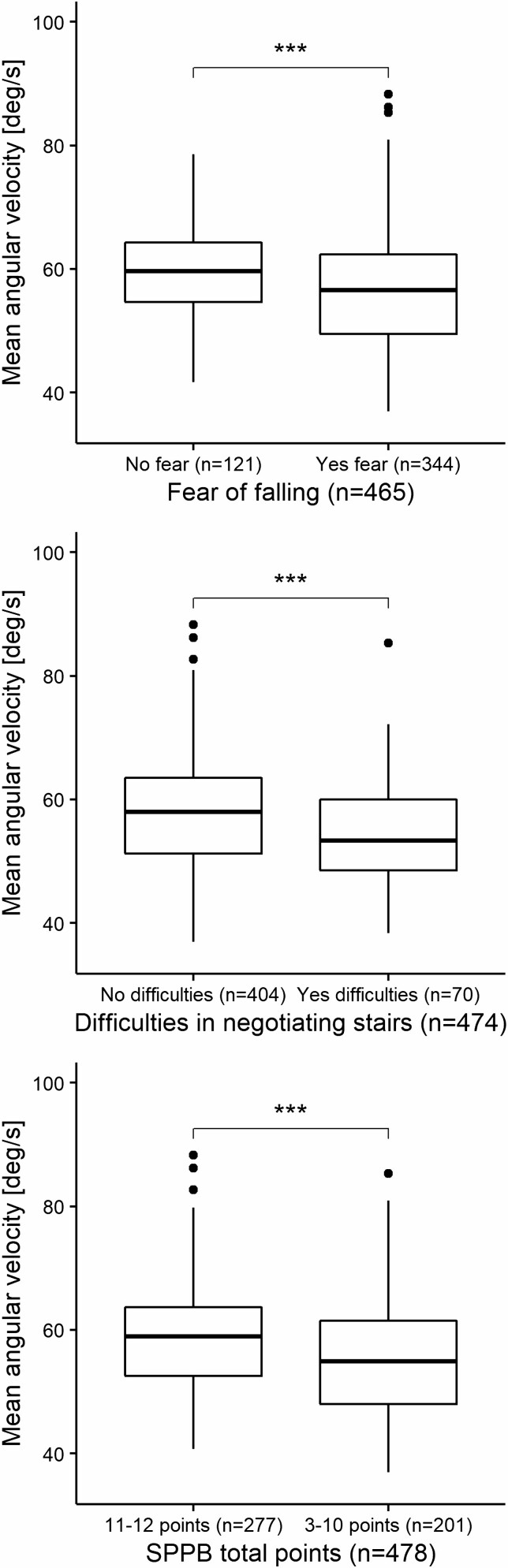
STS transitions mean angular velocity group comparisons between self-reported fear of falling, difficulties in negotiating stairs, and lower extremity functional limitations in free-living environment. Independent-Samples (unpaired) Mann–Whitney U Test (Wilcoxon rank-sum). ***p < .001 (2-sided). STS = sit-to-stand.

**Figure 3.**
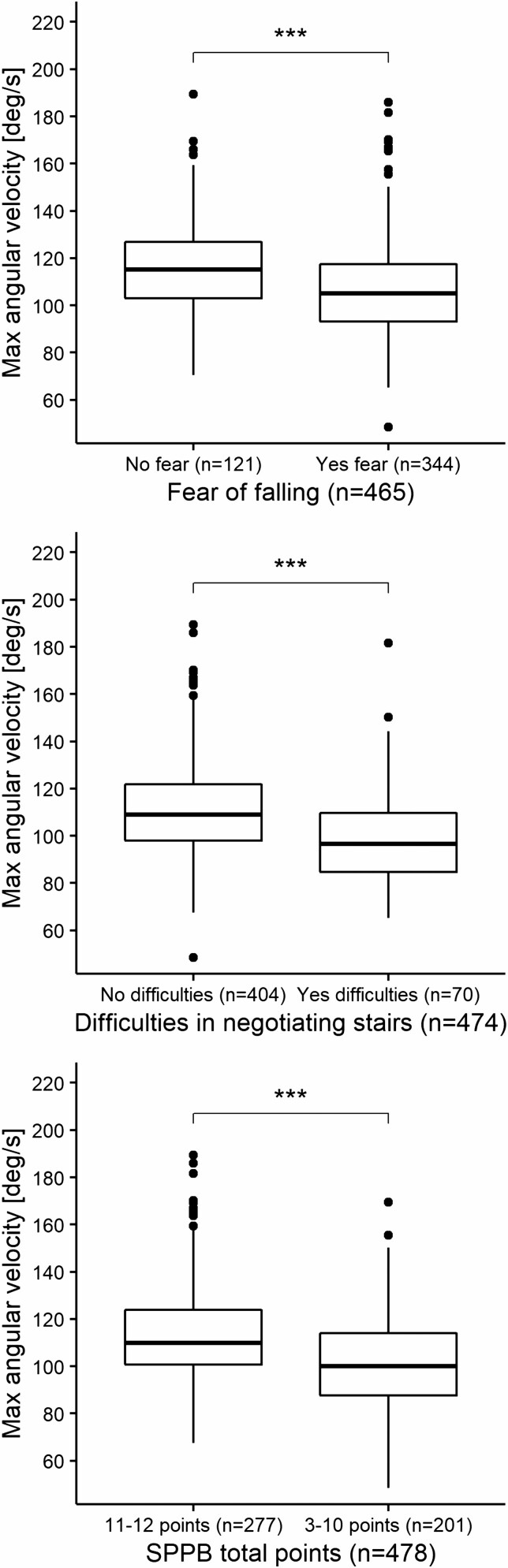
STS transitions maximal angular velocity group comparisons between self-reported fear of falling, difficulties in negotiating stairs, and lower extremity functional limitations in free-living environment. Independent-Samples (unpaired) Mann–Whitney U Test (Wilcoxon rank-sum). ***p < .001 (2-sided). STS = sit-to-stand.

Individuals who feared falling showed 15.8% fewer STS transitions (*p* < .001) and had 5.5% lower STS mean angular velocity (*p* < .001) and 8.9% maximal angular velocity (*p* < .001) in free-living conditions compared to individuals who reported no fear of falling Figure (1–3). Furthermore, individuals who reported difficulties with stair negotiation had 16.8% fewer STS transitions (*p* < .01) and had 6.9% lower STS mean angular velocity (*p* < .001) and 10.0% maximal angular velocity (*p* < .001) than individuals who reported no difficulties with stair negotiation Figure (1–3). Individuals who have lower extremity functional limitations according to SPPB total score showed 10.8% fewer STS transitions (*p* < .01) and had 5.8% lower STS mean velocity (*p* < .001) and 10.1% maximal angular velocity (*p* < .001) in free-living conditions compared to individuals who do not have lower extremity functional limitations Figure (1–3). Sensitivity analyses where we ran the sexes and ages independently indicated no effect of sex and age in the results.

## Discussion

The primary finding of the present study was that the volume and intensity of free-living STS transitions based on thigh-worn accelerometry were positively associated with lower-extremity performance, and negatively associated with lower extremity functional limitations, self-reported fear of falling, and difficulties in negotiating stairs among community-dwelling older people. Furthermore, the volume and intensity of STS transitions in free-living environment were lower in older age groups and differed between sexes. This study expands our understanding of free-living of physical activity by describing one of the most common specific daily activity movement and determining its intensity in community-dwelling 75-, 80-, and 85-year-old men and women. Assessing STS transitions may be a good daily performance indicator in future studies when monitoring older adults’ physical functioning in a free-living environment.

The number of STS transitions in the present study (women 42.8–34.4 transitions/day, men 50.4–41.2 transitions/day) is congruent with previously published results. Bohannon (2015) stated in his review that the average number of STS transitions are at least 45 per day among most community-dwelling individuals (1). In addition, Pickford and colleagues (2019) studied the mean angular velocity of STS transitions in 61.0 ± 10.1 years of age group and reported higher angular velocity values (70.7 ± 52.2 degree/s) compared to the present study (women 57.6–52.3 degree/s, men 60.6–55.2 degree/s). This is in line with the expected age-related decline in functional performance (36). In the current study, the volume and intensity of STS were lower with advancing age. This is in line with the higher number of STS transitions in younger people (71 ± 4 years) living at home compared to older people (87 ± 7 years) living in an older adult care facility (37). However, the sex difference observed in this study between STS intensity or number of transitions has not been studied in free-living conditions, although it has been found that STS transitions performance in the laboratory (5×STS time) decreases with age more slowly in women than men (23,38).

The associations between lower-extremity performance and number of STS transitions in the current study were relatively weak. Ryan et al. reported no significant (*r* = −0.12, *p* = .47) association between 5×STS test and free-living STS events in people with chronic low back pain (39). In addition, the tertile group comparisons in the present study indicated that the number of STS transitions did not seem to be very dependent on lower-extremity performance. This may suggest that the number of STS transitions is more related to the individual and environmental factors than laboratory-measured lower-extremity capacity, as noted for physical activity (40), especially when capacity does not limit STS transition in free-living environment. Furthermore, the intensity of the STS transition performance, as indicated by movement velocity, is influenced by multiple physiological and psychological processes rather than lower-extremity strength (23). Although the above-mentioned factors primarily affect STS transitions intensity, these factors may also have an effect on the number of STS transitions in the free-living conditions.

Knee extension force was more strongly related to the mean and maximal angular velocity of the STS transitions than to the number of STS transitions, indicating that maximal angular velocity, in particular might be a better representation of the capacity of the lower extremities. This is further confirmed by the tertile comparisons. In addition, a pairwise comparison of tertiles showed that the weakest tertile (T1) of mean or maximal angular velocity differed on lab-based lower-extremity performance from the other tertiles (T2 and T3), while no differences were found between tertiles T2 and T3. This could suggest that the mean and maximum angular velocities of the STS transitions only begin to decline when capacity is significantly impaired. Altogether, this could indicate that above a certain level of capacity, the intensity of the free-living STS transition does not increase linearly, which is well in line previously reported curvilinear relationship between knee extension force and 5×STS time (41). Therefore, future studies should investigate whether it is possible to identify cutoff points for the number of STS transitions and the angular velocities that would predict a lack of functional capacity.

To the authors’ best knowledge, the association between the knee extensor force and the angular velocity of STS transitions in a free-living environment has not been previously reported. Corrigan and Bohannon (2010) found a moderate correlation between knee extension force measured with a hand-held dynamometer and the duration of a single maximal STS transition performed in a laboratory, which is well in line with the correlation between knee extension force and maximal angular velocity of observed in the presents study. The association between 5×STS and STS transitions intensity was low. However, intensity quantified in this study (angular velocity) assessed only the STS phase, whereas 5×STS completion time also includes the stand-to-sit phase and any pauses between phases (18), which may differ between participants. To the best of our knowledge, the association between STS phase mean angular velocity and 5×STS completion time has not been reported, but a moderate association has been reported when comparing 5×STS (stopwatch) to the vertical velocity of the STS phase (42) which could be considered a comparable variable to the angular velocity.

Individuals who reported fear of falling presented a lower number, mean and maximal angular velocity of STS transitions compared to individuals who did not report fear of falling. According to the sensitivity analysis, no sex difference was observed. These results are well in line with the previously published results. Hornyak et al. (2013) have previously reported that fear of falling is related to total daily physical activity (43) and the number of STS transitions measured by accelerometer was weakly (*r* = −0.11, *p* = .009) associated with fear of falling (Fall Efficacy Scale-International, FES-I) in free-living conditions (44). In addition, concerns about falling have been found to be associated with low number of STS transitions among community-dwelling older men and women (45). Exploring difficulties in stair negotiation led to similar findings. Stair negotiating is one of the more demanding free-living activities older people engage with (46), but stair negotiation is challenging to identify from free-living accelerometry recordings (12). Given this challenge and the link between stair negotiation difficulties and STS transitions, examining the latter in free-living conditions seems both feasible and clinically relevant.

The decline in lower-extremity performance begins in middle-age, however, the decrements in physical capacity can be masked up to the age of 60–70 in submaximal activities such as walking (47). As STS transitioning requires a relatively high proportion (at least compared to walking) of the maximal force, we postulate that quantifying free-living STS transitions intensity among older adults could prove to be a sensitive indicator of future constraints in the ability to perform activities of daily living. In particular, the maximum angular velocity of STS transitions can describe a performance reserve that entails the ability to vary transition performance intensity, in the same way as walking speed reserve (48). In addition, the number of STS transitions have previously been used for monitoring frailty status (44). Following a similar line of reasoning, free-living STS transitions could also be linked to fall risk, although all these hypotheses would need to be tested with prospective study designs.

Some limitations need to be kept in mind when interpreting the findings. Firstly, we demonstrated that identification and intensity quantification of STS transitions is possible based on thigh-worn accelerometer data in free-living conditions. However, validity (ie, does the method measure what it purports to measure) could only be examined for STS transitions identification (Supplementary File), while the validity of the intensity quantification should be addressed in future studies. Moreover, the algorithm can only be applied to thigh-worn accelerometers sampling 3-dimensional accelerations. Nevertheless, the present results serve as an early indication of face validity (ie, are the values created by the method congruent with some relevant other measure) for intensity quantification. Secondly, the test–retest reliability of detection and intensity quantification in free-living conditions still remains to be established. To the best of our knowledge, reliability has only been evaluated in the laboratory environment (49). Third, the algorithm is only able to identify the first repetition of the multi-STS set (caused by the 2 seconds stationary epoch prior to a STS requirement) and therefore cannot be directly used to identify for example, the result in the 5×STS. The algorithm could be modified for use as an instrumented 5×STS assessment tool by removing the stationarity requirement from the algorithm. The stationarity requirement is necessary in free-living conditions to prevent false positives due to for example, cycling or walking. Fourth, using the arms during STS transitions cannot be controlled in the free-living environment. This can lead to misinterpretations, especially in determining the intensity, because STS transitions performed without using the arms have been found to have a slightly stronger association with STS performance than if the use of the arms is allowed in the laboratory environment (21). Finally, although the sample population was relatively old and based on a population representative sample, it is well established that those who volunteer for such monitoring are in better physical health or less frail compared to those who do not (26). Therefore, the findings may not be generalizable to the older population at large. On the other hand, the sample was based on a population representative sample.

The strength of this study can be considered a relatively large sample of community-dwelling participants. In addition, this study included multiple days (3–7 days) accelerometry recording, which is thought to be sufficient for assessing activity patterns (50). The strength of the study is also the versatile tests of physical performance performed in the laboratory (knee extension force) and at home (5×STS). In addition, the study protocol includes comprehensive questionnaires to assess participation limitations such as difficulties in negotiating stairs.

## Conclusion

Free-living STS volume and intensity were positively associated with higher lower-extremity performance and negatively associated with lower extremity functional limitations, self-reported fear of falling, and stair negotiation difficulties. The number and mean and maximal velocity of STS transitions in free-living situations was lower with advancing age and differed between sexes. The intensity of STS transitions was more strongly related to lower-extremity performance than the number of STS transitions. Due to the strength-demanding nature of transitioning from sitting to standing, we hypothesize that the proposed free-living STS transition quantification may enable identifying those at risk of future limitations in daily activities.

## Supplementary Material

Supplementary data are available at *The Journals of Gerontology, Series A: Biological Sciences and Medical Sciences* online.

glac071_suppl_Supplementary_Appendix

## Data Availability

After completion of the study, data will be stored at the Finnish Social Science Data Archive without potential identifiers (open access). Until then, pseudonymized data sets are available to external collaborators subject to agreement on the terms of data use and publication of results. To request the data, please contact Professor T.R. (taina.rantanen@jyu.fi).

## References

[1] Bohannon RW . Daily sit-to-stands performed by adults: a systematic review. J Phys Ther Sci. 2015;27(3):939–942. doi:10.1589/jpts.27.939 25931764 PMC4395748

[2] Guralnik J , Ferrucci L, Simonsick E, Salive M, Wallace R. Lower-extremity function in persons over the age of 70 years as a predictor of subsequent disability. N Engl J Med. 1995;332(9):556–562. doi:10.1056/nejm199503023320902 7838189 PMC9828188

[3] Nevitt MC , Cummings SR, Hudes ES. Risk factors for injurious falls: a prospective study. J Gerontol. 1991;46(5):164–170. doi:10.1093/geronj/46.5.m164 1890282

[4] Campbell AJ , Borrie MJ, Spears GF. Risk factors for falls in a community-based prospective study of people of 70 years and older. J Gerontol. 1989;44(4):M112–M117. doi:10.1093/geronj/44.4.m112 2738307

[5] Guralnik J , Simonsick E, Ferrucci L, et al A short physical performance battery assessing lower extremity function: association with self-reported disability and prediction of mortality and nursing home admission. J Gerontol. 1994;49(2):M85–M94. doi:10.1093/geronj/49.2.M85 8126356

[6] Giannouli E , Bock O, Mellone S, Zijlstra W. Mobility in old age: capacity is not performance. Biomed Res Int. 2016:2016:3261567. doi:10.1155/2016/3261567 PMC4789440 27034932

[7] VanLummel R , Walgaard S, Pijnappels M, et al Physical performance and physical activity in older adults: associated but separate domains of physical function in old age. Reddy H, toim. PLoS One. 2015;10(12):e0144048. doi:10.1371/journal.pone.0144048 26630268 PMC4667847

[8] Beard JR , Jotheeswaran AT, Cesari M, Araujo De Carvalho I. The structure and predictive value of intrinsic capacity in a longitudinal study of ageing. BMJ Open. 2019;9(11):e026119. doi:10.1136/bmjopen-2018-026119 PMC6830681 31678933

[9] Webber SC , Porter MM, Menec VH. Mobility in older adults: a comprehensive framework. Gerontologist. 2010;50(4):443–450. doi:10.1093/geront/gnq013 20145017

[10] Schrack JA , Cooper R, Koster A, et al Assessing daily physical activity in older adults: unraveling the complexity of monitors, measures, and methods. J Gerontol A Biol Sci Med Sci. 2016;71(8):1039–1048. doi:10.1093/gerona/glw026 26957472 PMC4945889

[11] Crowley P , Skotte J, Stamatakis E, et al Comparison of physical behavior estimates from three different thigh-worn accelerometers brands: a proof-of-concept for the prospective physical activity, sitting, and sleep consortium (ProPASS). Int J Behav Nutr Phys Act. 2019;16(1):1–7. doi:10.1186/s12966-019-0835-0 31419998 PMC6697962

[12] Skotte J , Korshøj M, Kristiansen J, Hanisch C, Holtermann A. Detection of physical activity types using triaxial accelerometers. J Phys Act Health. 2014;11(1):76–84. doi:10.1123/jpah.2011-0347 23249722

[13] Vähä-Ypyä H , Husu P, Suni J, Vasankari T, Sievänen H. Reliable recognition of lying, sitting, and standing with a hip-worn accelerometer. Scand J Med Sci Sport. 2018;28(3):1092–1102. doi:10.1111/sms.13017 29144567

[14] Janssen W , Bussmann J, Horemans H, Stam H. Validity of accelerometry in assessing the duration of the sit-to-stand movement. Med Biol Eng Comput. 2008;46(9):879–887. doi:10.1007/s11517-008-0366-3 18626677

[15] Vissers MM , Bussmann JBJ, de Groot IB, Verhaar JAN, Reijman M. Walking and chair rising performed in the daily life situation before and after total hip arthroplasty. Osteoarthr Cartil. 2011;19(9):1102–1107. doi:10.1016/j.joca.2011.06.004 21723401

[16] Van Roie E , Van Driessche S, Huijben B, Baggen R, Van Lummel RC, Delecluse C. A body-fixed-sensor-based analysis of stair ascent and sit-to-stand to detect age-related differences in leg-extensor power. PLoS One. 2019;14(1):e0210653. doi:10.1371/journal.pone.0210653 30653542 PMC6336282

[17] Zijlstra W , Bisseling RW, Schlumbohm S, Baldus H. A body-fixed-sensor-based analysis of power during sit-to-stand movements. Gait Posture. 2010;31(2):272–278. doi:10.1016/j.gaitpost.2009.11.003 19963386

[18] Van Lummel R , Walgaard S, Maier A, Ainsworth E, Beek P, Van Dieën J. The instrumented sit-to-stand test (iSTS) has greater clinical relevance than the manually recorded sit-to-stand test in older adults. PLoS One. 2016;11(7):1–16. doi:10.1371/journal.pone.0157968 PMC4938439 27391082

[19] Pickford CG , Findlow AH, Kerr A, et al Quantifying sit-to-stand and stand-to-sit transitions in free-living environments using the activPAL thigh-worn activity monitor. Gait Posture. 2019;73(May):140–146. doi:10.1016/j.gaitpost.2019.07.126 31325738

[20] Corrigan D , Bohannon RW. Relationship between knee extension force and stand-up performance in community-dwelling elderly women. Arch Phys Med Rehabil. 2001;82(12):1666–1672. doi:10.1053/apmr.2001.26811 11733880

[21] Eriksrud O , Bohannon RW. Relationship of knee extension force to independence in sit-to-stand performance in patients receiving acute rehabilitation. Phys Ther. 2003;83(6):544–551. doi:10.1093/ptj/83.6.544 12775200

[22] Martha R , Janelle K, Leigh A, Jordan C, Dulce F, Alyssa T. Functional predictors of stair-climbing ability in older adults. MOJ Gerontol Geriatr. 2017;1(5):115–118. doi:10.15406/mojgg.2017.01.00025

[23] Lord S , Murray S, Chapman K, Munro B, Tiedemann A. Sit-to-stand performance depends on sensation, speed,. balance, and psychological status in addition to strength in older people. J Gerontol A Biol Sci Med Sci. 2002;57(8):M539–M543. doi:10.1093/gerona/57.8.M539 12145369

[24] Bohannon RW . Knee extension strength and body weight determine sit-to-stand independence after stroke. Physiother Theory Pract. 2007;23(5):291–297. doi:10.1080/09593980701209428 17934969

[25] Tiedemann A , Shimada H, Sherrington C, Murray S, Lord S. The comparative ability of eight functional mobility tests for predicting falls in community-dwelling older people. Age Ageing. 2008;37(4):430–435. doi:10.1093/ageing/afn100 18487264

[26] Rantanen T , Saajanaho M, Karavirta L, et al Active aging―resilience and external support as modifiers of the disablement outcome: AGNES cohort study protocol. BMC Public Health. 2018;18(1):1–21. doi:10.1186/s12889-018-5487-5 PMC5930766 29716566

[27] Portegijs E , Karavirta L, Saajanaho M, Rantalainen T, Rantanen T. Assessing physical performance and physical activity in large population-based aging studies: home-based assessments or visits to the research center? BMC Public Health. 2019;19(1):1–16. doi:10.1186/s12889-019-7869-8 31775684 PMC6882080

[28] Vähä-Ypyä H , Vasankari T, Husu P, Suni J, Sievänen H. A universal, accurate intensity-based classification of different physical activities using raw data of accelerometer. Clin Physiol Funct Imaging. 2015;35(1):64–70. doi:10.1111/cpf.12127 24393233

[29] Löppönen A , Karavirta L, Portegijs E, et al Day‐to‐day variability and year‐to‐year reproducibility of accelerometer‐measured free‐living sit‐to‐stand transitions volume and intensity among community‐dwelling older adults. Sensors. 2021;21(18):6068. doi:10.3390/s21186068 34577275 PMC8471908

[30] Rantanen T , Era P, Heikkinen E. Physical activity and the changes in maximal isometric strength in men and women from the age of 75 to 80 years. J Am Geriatr Soc. 1997;45(12):1439–1445. doi:10.1111/j.1532-5415.1997.tb03193.x 9400552

[31] Guralnik J , Ferrucci L, Pieper C, et al Lower extremity function and subsequent disability: consistency across studies, predictive models, and value of gait speed alone compared with the short physical performance battery. J Gerontol A Biol Sci Med Sci. 2000;55(4):221–231. doi:10.1093/gerona/55.4.M221 PMC12149745 10811152

[32] Vasunilashorn S , Coppin AK, Patel KV, et al Use of the Short Physical Performance Battery Score to predict loss of ability to walk 400 meters: analysis from the InCHIANTI study. J Gerontol A Biol Sci Med Sci. 2009;64(2):223–229. doi:10.1093/gerona/gln022 19182232 PMC2655026

[33] Rantanen T , Portegijs E, Viljanen A, et al Individual and environmental factors underlying life space of older people―study protocol and design of a cohort study on life-space mobility in old age (LISPE). BMC Public Health. 2012;12(1):1–17. doi:10.1186/1471-2458-12-1018 23170987 PMC3534010

[34] Dipietro L , Caspersen CJ, Ostfeld AM, Nadel ER. A survey for assessing physical activity among older adults. Med Sci Sports Exerc. 1993;25(5):628–642. doi:10.1249/00005768-199305000-00016 8492692

[35] R Core Team. R: A language and environment for statistical computing. R foundation for statistical computing. Vienna, Austria. 2021. URL https://www.R-project.org/

[36] Hunter SK , Pereira XHM, Keenan KG. The aging neuromuscular system and motor performance. J Appl Physiol. 2016;121(4):982–995. doi:10.1152/japplphysiol.00475.2016 27516536 PMC5142309

[37] Egerton T , Brauer SG. Temporal characteristics of habitual physical activity periods among older adults. J Phys Act Health. 2009;6(5):644–650. doi:10.1123/jpah.6.5.644 19953842

[38] Butler AA , Menant JC, Tiedemann AC, Lord SR. Age and gender differences in seven tests of functional mobility. J Neuroeng Rehabil. 2009;6(1):1–9. doi:10.1186/1743-0003-6-31 19642991 PMC2741473

[39] Ryan CG , Gray H, Newton M, Granat MH. The convergent validity of free-living physical activity monitoring as an outcome measure of functional ability in people with chronic low back pain. J Back Musculoskelet Rehabil. 2008;21(2):137–142. doi:10.3233/BMR-2008-21210

[40] Rai R , Jongenelis MI, Jackson B, Newton RU, Pettigrew S. Factors influencing physical activity participation among older people with low activity levels. Ageing Soc. 2020;40(12):2593–2613. doi:10.1017/S0144686X1900076X

[41] Bohannon RW , Bubela DJ, Magasi SR, Wang YC, Gershon RC. Sit-to-stand test: performance and determinants across the age-span. Isokinet Exerc Sci. 2010;18(4):235–240. doi:10.3233/IES-2010-0389 25598584 PMC4293702

[42] Ejupi A , Brodie M, Gschwind YJ, Lord SR, Zagler WL, Delbaere K. Kinect-based five-times-sit-to-stand test for clinical and in-home assessment of fall risk in older people. Gerontology. 2015;62(1):118–124. doi:10.1159/000381804 26021781

[43] Hornyak V , Brach JS, Wert DM, Hile E, Studenski S, Vanswearingen JM. What is the relation between fear of falling and physical activity in older adults? Arch Phys Med Rehabil. 2013;94(12):2529–2534. doi:10.1016/j.apmr.2013.06.013 23816923 PMC4878685

[44] Parvaneh S , Mohler J, Toosizadeh N, Grewal GS, Najafi B. Postural transitions during activities of daily living could identify frailty status: application of wearable technology to identify frailty during unsupervised condition. Gerontology. 2017;63(5):479–487. doi:10.1159/000460292 28285311 PMC5561495

[45] Yu Shiu Z , Delbaere K, van Schooten KS. The relationship between concerns about falling and daily life activity in older men and women. J Aging Phys Act. 2021;30(2):217–224. doi:10.1123/japa.2020-0516 34407501

[46] Tikkanen O , Sipilä S, Kuula AS, Pesola A, Haakana P, Finni T. Muscle activity during daily life in the older people. Aging Clin Exp Res. 2016;28(4):713–720. doi:10.1007/s40520-015-0482-5 26526027

[47] Ferrucci L , Cooper R, Shardell M, Simonsick EM, Schrack JA, Kuh D. Age-related change in mobility: Perspectives from life course epidemiology and geroscience. J Gerontol A Biol Sci Med Sci. 2016;71(9):1184–1194. doi:10.1093/gerona/glw043 26975983 PMC4978365

[48] Middleton A , Fulk GD, Beets MW, Herter TM, Fritz SL. Self-selected walking speed is predictive of community walking behavior in older adults. Arch Phys Med Rehabil. 2015;96(10):e52. doi:10.1016/j.apmr.2015.08.172

[49] Regterschot GRH , Zhang W, Baldus H, Stevens M, Zijlstra W. Test-retest reliability of sensor-based sit-to-stand measures in young and older adults. Gait Posture. 2014;40(1):220–224. doi:10.1016/j.gaitpost.2014.03.193 24768083

[50] Pedersen ESL , Danquah IH, Petersen CB, Tolstrup JS. Intra-individual variability in day-to-day and month-to-month measurements of physical activity and sedentary behaviour at work and in leisure-time among Danish adults. BMC Public Health. 2016;16(1):1–9. doi:10.1186/s12889-016-3890-3 27914468 PMC5135790

