# Supplementary material for: Association Between Free-Living Sit-to-Stand Transition Characteristics, and Lower-Extremity Performance, Fear of Falling, and Stair Negotiation Difficulties Among Community-Dwelling 75 to 85-Year-Old Adults
Source: J Gerontol A Biol Sci Med Sci. 2022 Mar 21;77(8):1644–53. doi: 10.1093/gerona/glac071 (PMC9373963; doi:10.1093/gerona/glac071)
Supplement: glac071_suppl_Supplementary_Appendix [file glac071_suppl_supplementary_appendix.pdf]

Appendix A
Löppönen et al. 2022

STS algorithm's detection ability

Accelerations were recorded with a thigh-worn accelerometer in the laboratory testing session from (n = 782) AGNES study participants (1). The testing session included two known STS transitions records before 6 minutes walking test, where it was possible to unequivocally identify whether the transitions were detected by the STS detection algorithm. These two known STS transitions (Figure 1A) per laboratory testing sessions were used to develop the STS transition detection algorithm and to test the algorithm's detection ability.

Laboratory testing sessions were performed in 2017-2018. A roughly 45 min period from the laboratory session with two known sit-to-stand transitions was explored with the STS detection algorithm and the two STS transitions were manually identified from the data. The manual identification was used as the ground truth and compared to the detection algorithm.

Figure 1A. Sample of AGNES-study laboratory session (6MWT = 6 minutes walking test, APE = Angle Postural Estimation) two identifiable STS transitions marked with an arrow.

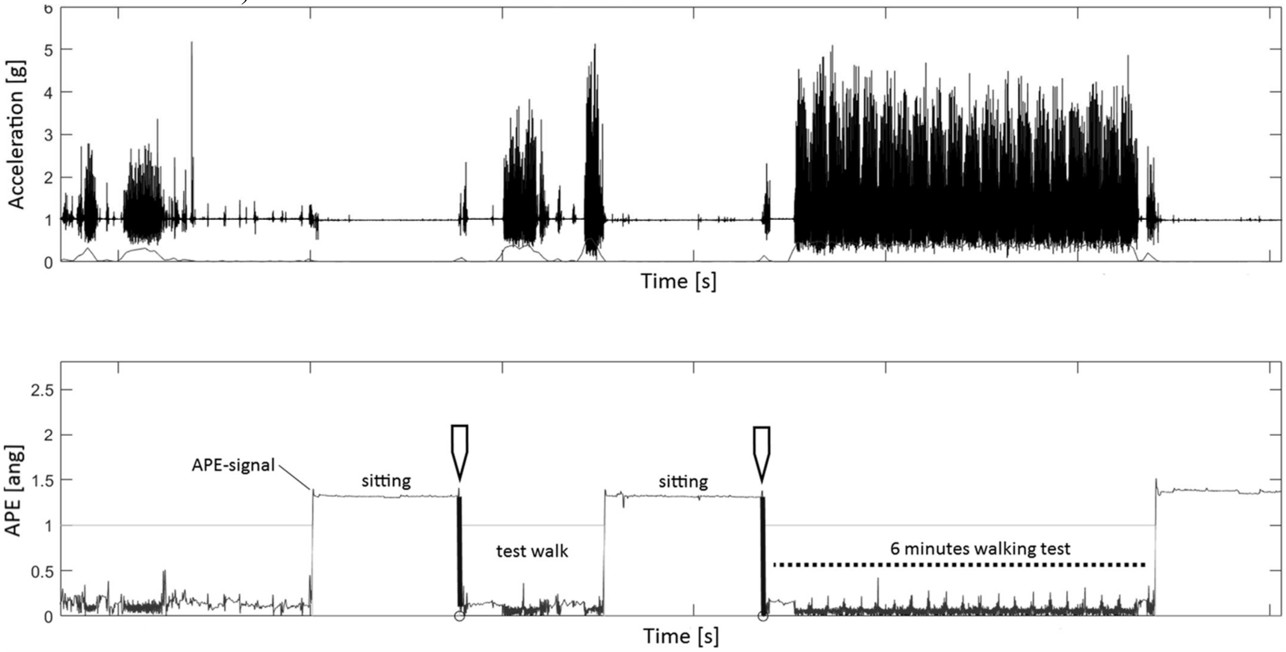

The overall accuracy of the STS transition detection algorithm was 93.3% in the laboratory environment. Overall algorithm correctly identified 1459 STS transitions (TP) and did not detect 105 (FN) of the known STS transitions. Splitting the detection accuracy by age and genders overall accuracy varied between 82.7% to 97.5% (Table A1). The lowest detection accuracy was found in the group of 85-year-old women in 82.7% and the highest detection accuracy in the group of 75-year-old men in 97.5%.

Table A1. The subgroup analysis of the STS transition detection algorithm.

	n	total STS	true positives TP	false negatives FN	overall accuracy
Men all	*339*	*678*	*650*	*28*	95,9 %
75 y	158	316	308	8	97,5 %
80 y	116	232	217	15	93,5 %
85 y	65	130	125	5	96,2 %
Women all	*443*	*886*	*809*	*77*	91,3 %
75 y	226	452	428	24	94,7 %
80 y	136	272	247	25	90,8 %
85 y	81	162	134	28	82,7 %
**Total all**	**782**	**1564**	**1459**	**105**	**93.3 %**

Detection considerations

Although the STS transition detection accuracy of the algorithm was very good and comparable to previously reported STS transition detection algorithms (2,3), we wish to highlight the conditions in which the algorithm failed. The greatest inaccuracy of the STS detection algorithm relates to occasions where the participants thigh was not stationary prior to the STS transition. Movement can be caused e.g., by wiggling or trembling of the foot (figure A2).

Figure A2. Identification of sit-to-stand (STS) transition based on an Angle for Postural Estimation (APE). A= True positive (TP) correctly detected, B= False negative (FN) detection failed (Y-axis = APE, X-axis = time).

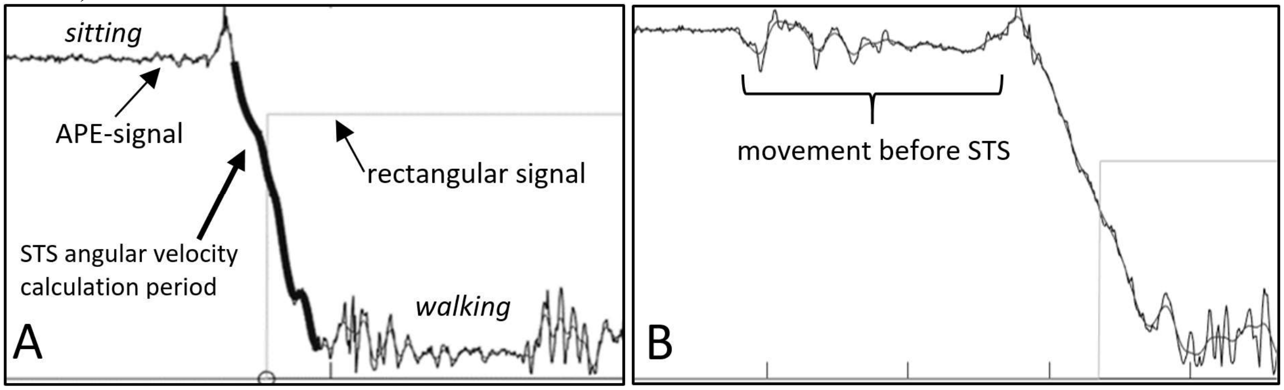

Additionally, the accelerometer is only capable of indicating the postural angle of the thigh. Consequently, incorrect interpretations may arise, for example, in situations where the thigh is extended in the absence of a sit-to-stand transition, or if horizontal acceleration is high enough (i.e. ≥ 1 g horizontal, which could arise e.g. due to the centripetal force in negotiating a change of direction) to appear as a change in posture as defined by the algorithm. Finally, the thigh is required to be close to horizontal (i.e., postural angle more than 65 deg) at the initiation of the STS transition. This makes detecting standing up from e.g., a saddle chair, medicine ball or other relatively high seats likely to fail.

References

1. Rantanen T, Saajanaho M, Karavirta L, et al. Active aging - Resilience and external support as modifiers of the disablement outcome: AGNES cohort study protocol. *BMC Public Health*. 2018;18(1):1-21. doi:10.1186/s12889-018-5487-5.
2. Pham MH, Warmerdam E, Elshehabi M, et al. Validation of a lower back “wearable”-based sit-to-stand and stand-to-sit algorithm for patients with Parkinson’s disease and older adults in a home-like environment. *Front Neurol*. 2018;9(AUG). doi:10.3389/fneur.2018.00652.
3. Rodríguez-Martín D, Samà A, Pérez-López C, Català A. Identification of Sit-to-Stand and Stand-to-Sit transitions using a single inertial sensor. *Stud Health Technol Inform*. 2012;177:113-117. doi:10.3233/978-1-61499-069-7-113.
